# A Report on Some Experiments with Trional

**Published:** 1895-11

**Authors:** Theodore Beyer

**Affiliations:** From the Fourth Division of the Garrison Hospital, Vienna


### Translation.

### A REPORT ON SOME EXPERIMENTS WITH TRIONAL.

By DR. THEODORE BEYER.
From the Fourth Division of the Garrison Hospital, Vienna.

Translated from *Wiener Medizinische Blätter*, No. 25, 1895,
By P. J. ROSENHEIN, M. D., New York.

THE high commendations which trional has received induced my honored chief, Dr. A. Tschudi, to employ it in a series of cases. Although, in consequence of the small number of cases reported, it is not possible to give a positive decision in regard to all the different properties of trional, we are justified, nevertheless, in formulating some conclusions concerning the position which trional occupies toward other hypnotics, especially toward morphine. Of course, these conclusions are restricted within narrow limits; for the wards of a military hospital, which are occupied for the most part by patients suffering from internal diseases, are deficient in the numerous interesting cases of functional exhaustion of the nervous system and in the various physical affections attended with excitement and sleeplessness, in which the use of an active and innocuous hypnotic so frequently contributes to recovery. Those of our patients who required the hypnotic were individuals suffering from painful affections accompanied by sleeplessness, and also, quite frequently, persons afflicted with fatal diseases whose sufferings demanded relief, if but for a few hours. Morphine has long been known as a comforter of these unfortunates, and, hitherto, has not been equaled by any other hypnotic, and if, therefore, in the following reports cases occur in which the much milder, although very serviceable, trional proved inefficient, these cannot militate against our favorable estimate of this remedy.

A few words with regard to its manner of employment. The doses varied from 0.5 to 2.0 gm., according to requirements. The time of administration was always 9 P. M. Care was taken that the trional powder was always administered with a sufficient amount of fluid (soup, milk, wine). Attention was paid to the bowels, in consideration of the phenomena (diminution of the alkalinity of the blood, hematoporphyrinuria) which are observed after prolonged employment of its congener, sulfonal. It seems superfluous to adopt any special regulations of the diet. An abundant administration of citric acid in the form of the officinal potus acid citrici was ordered. Below we give short reports of the above-mentioned cases :

CASE I.—St. M., aged 24, recruit, suffering from incipient phthisis on both sides, was attacked by a left-sided pleurisy attended with rigor and high fever. The exudation increased quite rapidly, extending anteriorly to the clavicle and produced marked symptoms of compression. By puncture, 1 litre of pus, containing streptococci was evacuated. This was followed by a progressive loss of strength, recurring dyspnea and death after five days in collapse.

During the accumulation of the exudate, trional was administered for eight days in doses of 1.0 gm. for the relief of pains and sleeplessness. The effect was remarkably good ; up to the time that the exudate reached its highest level the patient slept well until morning, and expressed his satisfaction with the medication.

CASE II.—V. G., aged 16 years, hereditary predisposition, was admitted to the hospital with pronounced chronic phthisis on both sides. After several months' treatment, which was unable to prevent extension of the process, the patient began to be sleepless. Trional, 1.0 gm., was administered. The effect was surprisingly marked ; the patient not only slept the entire night, but until late in the morning ; he also passed the following day in a drowsy condition, and the following night in deep sleep. For this reason the dose was reduced to 0.5 gm. During almost two months the effect was very satisfactory ; the patient slept well and suffered from no after-effects. Not until the later stages of the disease, when the patient's vitality had become exhausted, was it found impossible to obtain the same favorable results.

CASE III.—A. K., aged 22, corporal, hereditarily predisposed, was admitted with commencing infiltration at the apices. Patient was much disturbed in his night's rest by cough, fever and night sweats and requested a hypnotic. During five days, doses of 1.0 gm. trional were administered, with the desired result, and patient passed his nights in quiet sleep. After the sixth dose its effect failed to occur and rest was only secured by internal administration of morphine. No further observations were made and patient returned to his home.

Case IV.—J. L., aged 21, soldier; admitted with bilateral tuberculosis at the apices, and echinococcus of the liver. Patient decreased visibly in strength and a pleural exudation developed on the right side, with marked dyspnea and insomnia. As radical treatment seemed unavailable trional in doses of 1.0 gm. was employed with good success for three weeks, with alleviation of his sufferings. Patient obtained rest at night and frequently slept until late in the morning.

Case V.—F. A., aged 22, soldier in the infantry, suffered from a severe diffuse febrile bronchitis, attended with violent cough, difficulty in breathing and sleeplessness. During the acme of the disease process trional 1.0 gm. was administered for five evenings. The result was satisfactory; patient had less cough and slept uninterruptedly for four to five hours.

Case VI.—A. F., aged 29, watchman, was admitted to the hospital with the same symptoms as above. Trional was administered in 1.0 gm. doses for fourteen days and manifested equally favorable effects; the patient enjoyed sleep of five to six hours' duration and passed the remainder of the night in an equally good condition.

Case VII.—J. W., aged 24, was admitted for the same disease. The restlessness and respiratory disturbances were especially marked; the percussion note was slightly dull over the lower lobe in consequence of accumulation of secretion. Here, also, trional, administered in 1.0 gm. doses for ten days, acted efficiently; patient slept uninterruptedly until three in the morning and sometimes later.

Case VIII.—F. Sch., aged 24, soldier in infantry, was admitted with a severe phlegmonous angina. There were present high fever, difficulty in swallowing and severe pains radiating over the face and neck. As the latter persisted, after an incision had been made, trional was administered in 1.0 gm. doses for eight days. The effect was admirable and patient slept undisturbed until the morning hours.

Case IX.—F. V., aged 24, corporal, also suffered from a phlegmonous angina of great severity. Here, also, the remedy was employed with excellent effect in 1.0 gm. doses for two nights.

Case X.—F. D., aged 29, sergeant, afflicted with articular rheumatism, acquired, during his sojourn at the hospital, a tonsillar abscess on the left side; severe pains and sleeplessness were present during several days. Trional, 1.0 gm., was administered for two evenings, and produced a brief sleep of one to two hours' duration. On the third evening the dose was increased to 2.0 gm., and produced sufficient sleep, although, on the following morning, the patient was in a disagreeable drowsy condition, so that the double dose had to be discontinued.

Case XI.—G. K., aged 22, soldier in infantry, was admitted with a typical pneumonia of the right lower lobe and marked diffuse bronchitis. During the stage of resolution, violent cough and sleeplessness occurred. As trional, 1.0 gm., proved inefficient, 2.0 gm. were administered, with a production of sleep from 9 P. M. to 2 A. M. The double dose, however, was badly borne, and the patient stated that he felt confused and suffered from violent headache and vertigo ; for this reason, trional was discontinued.

Case XII.—F. M., aged 23, soldier in infantry, was admitted with the symptoms of a violent gastro-enteritis attended with peritoneal irritation. Respiration was accelerated and superficial, and there was high fever, 30° to 40° C. Vomiting of material colored with bile, tympanites, marked tenderness of the abdomen and bloody diarrheal stools were present. As the patient was unable to sleep at night, trional in doses of 1.0 gm. was administered on six occasions, but without adequate effect, since the patient did not fall asleep until midnight. Under the other treatment his condition improved, so that the trional could be dispensed with.

Case XIII.—R. Sch., aged 24, cannoneer, was admitted with metastatic sarcoma of both lungs. There were present hemoptysis, due to compression, especially of the right lung, severe dyspnea and insomnia. Trional was employed on ten occasions ; doses of 0.5 gm. proved absolutely inefficient, and with larger doses, 1.0 to 2.0 gm., a brief sleep, of one to three hours' duration, could be obtained only twice. As alleviation of the distressing condition of the patient appeared urgently demanded, trional was replaced by the sovereign remedy, morphine.

Case XIV.—Th. W., aged 22, had sustained a rupture of the left drum membrane, in consequence of a blow on the ear, which was followed by a purulent otitis media, despite the most careful treatment. A severe febrile condition rapidly developed, with terrible unilateral headaches and continued insomnia. The suppuration made its way through the inner ear to the brain membranes ; facial paralysis with dimness of vision supervened, and soon the patient became delirious and later on unconscious, and succumbed in coma under marked febrile phenomena. For the relief of some of the symptoms, trional in doses of 1.0 gm. was employed without any effect, and the desired results could be obtained only by injections of morphine.

Case XV.—F. B., non-commissioned officer, suffering from chronic tuberculosis of both lungs, was admitted to the hospital with all the symptoms of a severe perityphlitis, which was associated with violent peritoneal irritation, fever, extreme painfulness of the abdomen and insomnia. Patient was treated symptomatically with injections of morphine, but soon became habituated to it to such an extent that he was unable to obtain any rest without it, and, when refused, repeatedly requested help from the inspecting physicians. The sleeplessness increased at a subsequent period, when a left-sided pleurisy, with marked exudation, violent lancinating pains and dyspnea made its appearance, and larger doses of morphine had to be resorted to. In place of this drug, trional was administered in doses of 1.5 gm. The effect was very variable. On several occasions the patient slept quite well, with few interruptions, but more frequently he passed disturbed and, for the most part, sleepless nights. After the patient had used sixteen doses, with an interval of three days, he became greatly constipated and manifested a decided repugnance towards trional, so that its continued administration appeared inadvisable, and, at the urgent request of the patient, recourse was again had to hypodermics of morphine. The autopsy showed, as a cause of the circumscribed peritonitis, a perforation of the vermiform appendage by a concretion of the size of a bean and yellowish brown color.

The observations on trional which have appeared up to the present time, and are given in detail in the work of Dr. Otto Bakofen, are derived, for the most part, from psychiatric clinics. The authors concur unanimously in regarding trional as an excellent sedative and hypnotic, and recommend its employment in the various forms of neurasthenia, psychoses attended with moderate excitement, and in organic affections of the brain. On the other hand, in the sleeplessness due to bodily pains, negative results have been frequently reported.

The above-mentioned series of observations, as will readily appear from what has been before said, relate exclusively to cases of the latter kind, and, notwithstanding this, the results in general must be considered satisfactory. In Cases I. to IX., which were attended with considerable somatic disturbances, the effect of the remedy was most admirable. A deep and undisturbed sleep, of about five to six hours' duration, ensued from a quarter to half an hour after its administration. There were no disagreeable sequelæ and the sleep resembled perfectly the normal. In Cases X. and XI., larger doses (2.0 gm.) were required for obtaining a hypnotic effect, and here the sequelæ mentioned by several authors were observed. A disagreeable feeling of drowsiness, headache, vertigo and a sensation of reeling were experienced, while nausea, vomiting and tinnitus were never observed. Aside from these comparatively slight disturbances of the general condition, no interference with the functions of vital organs occurred. In Cases XII. to XV., comprising patients severely ill, in which the employment of trional, as would appear from previous reports, promised but little, the remedy proved ineffective. The patients slept little, or not at all, and morphine had to be resorted to.

Briefly formulated, the above series of experiments demonstrates that, in cases of insomnia due to moderate bodily disturbances, trional may be employed with advantage. If properly administered in proper doses, no disagreeable after-effects or sequelæ and no habituation will be observed. On the other hand, in severe cases it is utterly unable to replace morphine.

